# Mechanical Modeling of Healthy and Diseased Calcaneal Fat Pad Surrogates

**DOI:** 10.3390/biomimetics4010001

**Published:** 2019-01-03

**Authors:** Arnab Chanda, Stephen McClain

**Affiliations:** 1 Department of Bioengineering, University of Pittsburgh, PA 15213, USA; 2 Department of Aerospace Engineering and Mechanics, University of Alabama, AL 35401, USA; scmcclain@gatech.edu; 3 Department of Biomedical Engineering, Georgia Institute of Technology, GA 30332, USA

**Keywords:** foot, diabetes, calcaneal, heel, plantar fasciitis, custom orthotic

## Abstract

The calcaneal fat pad is a major load bearing component of the human foot due to daily gait activities such as standing, walking, and running. Heel and arch pain pathologies such as plantar fasciitis, which over one third of the world population suffers from, is a consequent effect of calcaneal fat pad damage. Also, fat pad stiffening and ulceration has been observed due to diabetes mellitus. To date, the biomechanics of fat pad damage is poorly understood due to the unavailability of live human models (because of ethical and biosafety issues) or biofidelic surrogates for testing. This also precludes the study of the effectiveness of preventive custom orthotics for foot pain pathologies caused due to fat pad damage. The current work addresses this key gap in the literature with the development of novel biofidelic surrogates， which simulate the in vivo and in vitro compressive mechanical properties of a healthy calcaneal fat pad. Also, surrogates were developed to simulate the in vivo mechanical behavior of the fat pad due to plantar fasciitis and diabetes. A four-part elastomeric material system was used to fabricate the surrogates, and their mechanical properties were characterized using dynamic and cyclic load testing. Different strain (or displacement) rates were tested to understand surrogate behavior due to high impact loads. These surrogates can be integrated with a prosthetic foot model and mechanically tested to characterize the shock absorption in different simulated gait activities, and due to varying fat pad material property in foot pain pathologies (i.e., plantar fasciitis, diabetes, and injury). Additionally, such a foot surrogate model, fitted with a custom orthotic and footwear, can be used for the experimental testing of shock absorption characteristics of preventive orthoses.

## 1. Introduction

**Figure 1:**
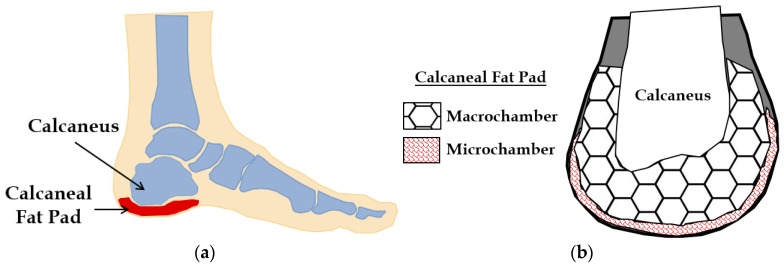
(**a**) The foot with calcaneus bone and the calcaneal fat pad. (**b**) Calcaneus and calcaneal fat pad architecture.

The foot is the most distal part of the human leg and functions to transmit our weight to the ground, balance our posture, and assist in locomotion [1]. During the gait cycle, the heel is the first point of contact between the body and the ground, and is the primary absorber of the ground reaction force. The calcaneus, or heel bone, is supported and cushioned by the calcaneal fat pad that functions as a shock absorber (see Figure 1a). The average heel fat pad is 18 mm thick, and is composed of honeycombed fat globules organized into a bilayer: the macrochamber and microchamber (Figure 1b). These globules are held together by a collagenous septum. The organization of the fat pad into macrochambers and microchambers affects the nonlinear anisotropic material behavior of the heel pad [2]. Compared to macrochambers, the microchamber is a more superficial layer, which is known to be nearly incompressible [3]. Microchambers have been found to experience very little deformation under loading conditions and have a modulus of elasticity ten times greater than that of macrochambers [3]. The macrochamber, on the other hand, undergoes significant deformation under loading, and is majorly responsible for the compressibility and mechanical properties of the heel pad. Ledoux et al. [4] demonstrated that the distinct mechanical properties of the heel pad can be majorly attributed to the unique structure of the calcaneal fat and somewhat to the other fatty layers of the foot (subphalangeal and submetatarsal). The heel pad, when subjected to variable loads, was found to exhibit a prolonged relaxation time, accompanied with an increase in the modulus of elasticity and a decrease in the energy dissipation. The force dissipating effects of the heel fat pad were observed to be critical for reducing the effect of loads absorbed by the calcaneus and surrounding structures. Also, the heel pad was found to slowly lose its elasticity and energy dissipating properties as a shock absorber due to age, obesity, and diabetic conditions [5]. The major contributing factor behind this occurrence was found to be adipose tissue atrophy and the degeneration of the collagenous septum that forms the honeycomb structure within the bilayer. Additionally, fat pad atrophy and reduced shock absorbance in the heel was observed to have a strong connection with incapacitating heel pain and diabetic ulceration [6].

In the literature, mechanical tests have been conducted on the calcaneal fat pad at varying strain rates [7,8,9,10,11] and frequencies [4] to characterize its mechanical properties. Strain rates corresponding to different loading conditions—standing [12], walking [8], running [13], and impact-loading [14]—have been simulated experimentally on the heel pad to understand its biomechanical behavior. Also, a few studies have been conducted to characterize the mechanical properties of fat pad in diseased conditions [15,16,17]. Wearing et al. [18] used dynamic radiographs to compare the in vivo stress versus strain response in the foot of cohort groups with and without plantar heel pain. Their findings suggest that there was no significant disparity between the peak stresses and strains, nor the loaded and unloaded heel pad thickness of the two groups. However, the results showed the energy dissipation ratio (EDR), calculated as the fraction of the area under the load versus deformation hysteresis plot and the area underneath the loading curve [10], to be roughly 20% less in the group with heel pain. Fontanella et al. [10] compressed in vivo the heel pad of a healthy 30 year-old woman at displacement rates of 0.8 and 1.96 mm/s by fastening the foot to a vertical plate and applying force to the heel with a piston, to a maximum deformation of 9 mm. Using the results of the load–displacement curves, the energy dissipation ratios at displacement rates 0.8 and 1.96 mm/s were calculated to be 0.25 ± 0.02 and 0.26 ± 0.03, respectively. Grigoriadis et al. [11] conducted quasi-static and dynamic tests on five cadaveric heel specimens in order to estimate subject-specific and average material properties of the calcaneal heel pad under compressive loads at different strain rates, by using an inverse finite element (FE) optimization algorithm.

To date, a plethora of surrogate models exist for soft tissues such as the calcaneal heel pad [19,20], which have been characterized using indentation systems (measuring external load–deformation responses) [20], imaging (for internal strain measurements) [21], and experienced palpatory testing [22]. These surrogates have been used in anthropomorphic test devices for the study of injury risks associated with the lower extremity due to blast loading [14,19,23,24] and high impact loadings sustained in vehicular crashes [25,26]. Also, surrogate models have been employed in several studies to characterize the effect of personal protection equipment [27], and orthotic interventions for injury prevention [28,29]. However, all such surrogates (composed of standard polyurethane and silicone-based elastomers), which have been characterized using average linear elastic material models [20], have mechanical properties widely different from the soft tissues with nonlinear properties [30,31]. Also, these surrogates are not based on in vivo and in vitro mechanical testing of soft tissues, leading to questions about their biofidelity and applicability in testing efforts related to the foot pain and custom orthosis development. 

Fabrication of biofidelic calcaneal fat pad surrogates—with accurate nonlinear mechanical properties at different strain (or displacement) rates, and tested under dynamic and cyclic loads—can be integrated with prosthetic foot models for mechanical testing and would allow for the investigation of fat pad shock absorption in different simulated gait activities, and due to varying fat pad material properties in foot pain pathologies (i.e., plantar fasciitis, diabetes, and injury). Additionally, such a foot surrogate model, fit with a custom orthotic and footwear, can be used for the experimental testing on the shock absorption characteristics of preventive orthoses. In this work—based on development of biofidelic tissue surrogates for skin [30,32,33,34,35,36,37,38], muscles [36,39,40,41], the pelvis [42,43,44,45,46], brain [30,32,47], and arteries [39,48,49]—novel biofidelic calcaneal fat pad surrogates were developed using a four-part elastomer-based material. The fabrication, testing, and characterization methodologies of these surrogates are presented in the upcoming sections, followed by the results and discussion, and conclusions. 

## 2. Materials and Methods

### 2.1. Surrogate Fabrication

**Figure 2:**
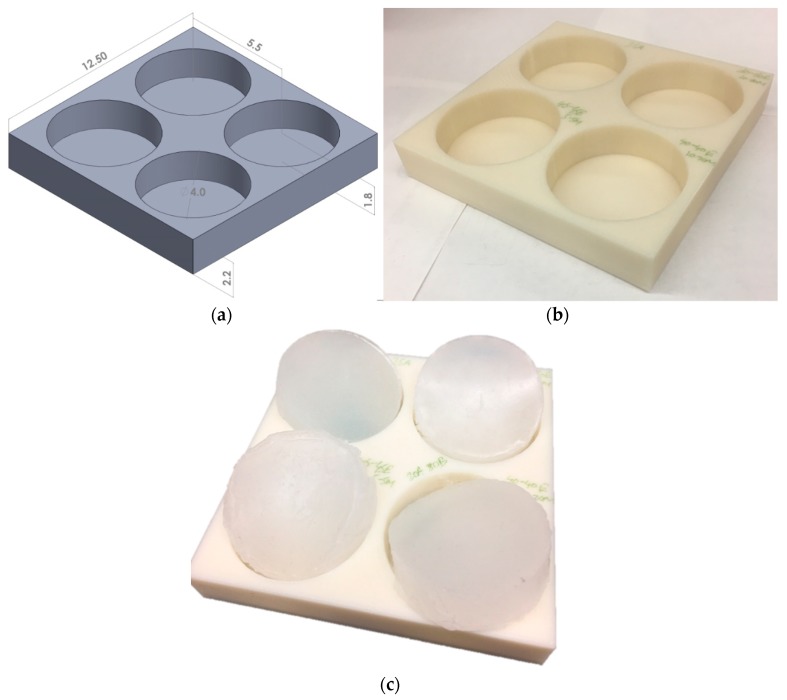
(**a**) Mold computer-aided design (CAD) image with all dimensions in cm, (**b**) 3D printed mold, and (**c**) some calcaneal fat pad surrogate test specimens.

For this study, a two-part elastomer material with shore hardness 10 (Ecoflex 0010, Smooth-On, Inc., Macungie, PA, USA) and another two-part elastomer with shore hardness 30A (Mold Star 30, Smooth-On, Inc.) were procured and mixed to obtain a four-part mixture. Each composition was prepared by precisely measuring the four parts of the elastomer using experimental measuring cups and an Ohaus Adventurer Pro precision weight measuring device (Ohaus Corporation, Parsippany, NJ, USA), followed by thorough mixing for 1 min and curing for approximately 3 h. To cast the liquid mixture in the shape and size of the calcaneal heel pad, a mold was designed using the Solidworks software (Dassault Systèmes, Vélizy-Villacoublay Cedex, France). This mold had four cylindrical chambers, each with a diameter of 40 mm and depth of 18 mm (Figure 2a), chosen based on the literature [16,50,51]. The overall dimension of the mold was 125 mm × 125 mm, with a square cross-section and a 22 mm depth. This design was exported as a stereolithographic (STL) file and three-dimensional (3D) printed at the University of Alabama 3D printing studio in the Rodgers Library using a Stratasys uPrint SE printer (Eden Prairie, MN, USA). Forty test specimens with elastomer compositions in the range of 5–35 wt % of part A (Shore 30A), 5–35 wt % of part B (Shore 30A), 65–95 wt % of part A (Shore 00-10), and 65–95 wt % of part B (Shore 00-10) were tested for surrogate fabrication. Figure 2 shows some test specimens and Table 1 summarizes the 15 different compositions tested, which resulted in distinguishable mechanical responses. These compositions were able to simulate the nonlinear compressive mechanical properties of all types of calcaneal fat pads tested in the literature [2,7,8,9,10,11,12,13,15,16,18,50,51]. 

### 2.2. Mechanical Testing

**Figure 3:**
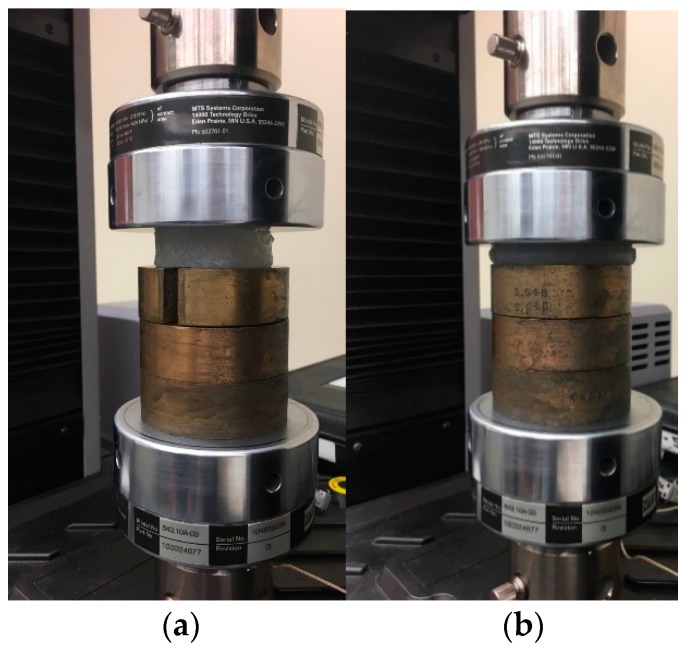
A dynamic compressive test at 0.8 mm/s strain rate for times (**a**) *t* = 0 s, and (**b**) *t* = 12 s.

Uniaxial compression tests were conducted on 40 test specimens using a materials testing machine (MTS Exceed Series 40, Model E42.503, Eden Prairie, MN, USA). Several considerations were adopted for testing of the soft surrogate materials. First, strain (or displacement) rates significantly affect the load response of soft materials [52] and this phenomenon has been observed in tissues [32], which necessitates the use of a specific strain (or displacement) rate so that results can be precisely compared with those from the literature. Second, the shape and size of the specimens may affect the test results [52]. Third, the load application direction can substantially change the test results due to specimen orientation and material anisotropy. To avoid such inconsistencies, standard size isotropic test specimens were employed in this study and results were compared with the literature for the same strain (or displacement) rate. Also, to ensure that the specimen surfaces are smooth and even, they were casted in molds with tight dimension tolerances, and checked for uniformity using Vernier calipers after each trial. Figure 3 shows a compression test performed on a test specimen. In this test setup, interlocking cylindrical copper weights (diameter of 60 mm and depth of 22 mm) were employed to raise the height of the sample, as the testing machine was designed to operate at a minimum safety gap of 40 mm. These weights were placed concentric with the compression plates and secured with dowel pins. Since the load cell was installed on the top compression plate, the raised stationary platform was assumed to not affect the mechanical test results. The effect of the end slot on the weights was unknown. An initial compliance test was conducted by running three trials at two displacement rates (0.8 and 1800 mm/s), with two weights (i.e., height of specimen base was 44 mm) and three weights (i.e., height of specimen base was 66 mm). The resulting load–displacement plots were compared. The results were similar for all tests with an average correlation index of 0.96. Therefore, it was concluded that the raised platform, and openings on the weights will not affect the study results. 

All tests were conducted at a constant displacement rate (or strain rate similar to those found in the literature), and a load cell was employed to quantify resisting forces. A test rate of 0.80 mm/s was selected to conduct dynamic compression tests (up to a maximum displacement of 9 mm) on the surrogates (each composition tested three times) and the results were compared with in vivo calcaneal fat pad tests (with the flat indenter or piston, which had the same size as that of the surrogates) in the literature [10,16]. Also, surrogates were identified to simulate the mechanical properties of the heel pad due to plantar heel pain and diabetes [16]. Additionally, a surrogate composition was identified which simulates the average mechanical properties of healthy calcaneal fat pad tested in the literature [10,16], and was named as the control surrogate. This control surrogate was subjected to cyclic loading and unloading tests (with ten test specimens, each for five cycles) at a test rate of 1.96 mm/s to understand its repeatability and to compare with in vivo cyclic tests in the literature [10]. All cyclic tests also included two initial idle trials, followed by the five cycles with a one-minute break between each trial. The displacement rate was kept constant for 4 s, and the maximum displacement and load values were set to 9 mm and 40 N, respectively, based on the literature [10]. Further tests were conducted at different displacement rates (1.96, 180, and 1800 mm/s) to compare the mechanical properties of the healthy control surrogate with that of in vitro (cadaveric) heel pad tests in the literature [11]. 

A potential limitation of in vitro tests with cadaveric tissue samples is its difference in mechanical properties from living tissues due to the nonconsideration of factors such as blood propulsion and hydration. As described in the literature [52], blood pressure has been shown to not affect the mechanical properties of the heel pad at high compression rates (>400 mm/s), and minimally affect tissue stiffness (<3%) at lower test rates. Also, the calcaneal heel pad has been observed to not differ in mechanical behavior with hydration (measured post-amputation samples compared with preserved samples) [53]. Unlike in vitro studies where the calcaneal fat tissue is the only component, in vivo studies involve the effects of the other tissue and joint components (such as the calcaneus bone) with different topologies within the entire limb. To consider the possible overall effect of such interactions, the biofidelic surrogates were developed based on controlled in vivo experiments in the literature [10], where the longitudinal axis of the foot was placed in line with the loading axis of the compression device, the piston and heel pad were assumed to have an almost flat surface-to-surface contact, and the foot was fixed with fasteners to restrict the movement of all possible joints. The only exceptions were the high displacement rate tests which are not feasible in vivo. Therefore, the surrogates tested at high displacement rates were compared with in vitro tests [52]. 

### 2.3. Material Modeling

Soft tissues and synthetic biomaterials can be characterized using hyperelastic material models, which are able to accurately model the nonlinear stress (σ) versus strain (ε) response due to compression [30,32,34,37,54,55,56,57,58]. Hyperelastic constitutive models are based on the strain energy function (ψ), which depends on the type of material [59,60]. The strain energy function is dependent on either the principal stretches ($\lambda_{1}$, $\lambda_{2}$, and $\lambda_{3}$, where λ=ε+1) or the Cauchy–Green tensor invariants ($I_{1}$, $I_{2}$, and $I_{3}$) [59,61,62], given by $I_{1}=\sum_{i=1}^{3}\lambda_{i}^{2}$, $I_{2}=\sum_{i,j=1}^{3}\lambda_{i}^{2}\lambda_{j}^{2}$, i≠j, and $I_{3}=\prod_{i=1}^{3}\lambda_{i}^{2}$. For this study, Yeoh’s model, which has been used previously to accurately characterize rubbers and tissues, was selected to conduct the characterization of the calcaneal heel pad surrogates at different displacement rates. In a uniaxial tension or compression test, Yeoh’s isotropic strain energy function is given by $\psi =c_{1}{\left[I_{1}-3\right]}_{}^{}+c_{2}{\left[I_{2}-3\right]}_{}^{2}+c_{3}{\left[I_{3}-3\right]}_{}^{3}$, where $c_{1}$, $c_{2}$, and *c*_3_ are the hyperelastic parameters. Applying uniaxial boundary conditions and assuming incompressibility, the stress–stretch relationship is given by $\sigma =2(\lambda^{2}-\frac{1}{\lambda })(c_{1}+2c_{2}[I_{1}-3]+3c_{3}{\left[I_{1}-3\right]}_{}^{2})$. 

It should be mentioned here that the hyperelastic model chosen does not take into account displacement rate effects, and thus a separate set of hyperelastic parameters would represent the mechanical behavior of the same surrogate composition tested at two different test rates. Advanced viscoelastic characterization models are investigated in the future to characterize the displacement or strain rate effects of these surrogates.

### 2.4. Data Analysis

The engineering stress versus stretch results recorded from the mechanical tests were analyzed using Microsoft Excel software (Microsoft Office Professional Plus 2016, Microsoft Corporation, Albuquerque, NM, USA). The average and standard deviation of stress-stretch were computed and plotted across the number of tests conducted for the calcaneal fat pad surrogates, for the three groups (healthy control, diabetes, and plantar heel pain) at 0.08 mm/s, cyclic tests on the healthy control group at 1.96 mm/s, and high strain rate tests on the healthy control group at 1.96, 180, and 1800 mm/s. The hyperelastic curve fitting was conducted in multiple steps. First, the stretch increment values from the experiments were substituted into the Yeoh’s hyperelastic stress-stretch equation (Section 2.3), along with any arbitrary values assigned for the curve-fit coefficients ($c_{1}$, $\;c_{2}$ and $c_{3}$). Second, the predicted stress values were subtracted from the experimental stress values for all stretch increments, and squared. Third, these squared values were added for all stretch increments and the minimum sum was computed using the MS Excel curve fit solver (as the best values of $c_{1}$, $\;c_{2}$ and $c_{3}$ were predicted). To determine the accuracy of the estimated curve-fit coefficients, an *R^2^* correlation quantity was computed between the experimental stress values and predicted stress values (with the estimated $c_{1}$, $\;c_{2}$ and $c_{3}$) across all stretches. All analyses were conducted with a significance level (*α*) of 0.05. For *R^2^* above a threshold of 0.95, the curve fit-coefficients were considered to be highly accurate.

## 3. Results and Discussion

The mechanical compression test results of the 15 surrogate compositions and their comparison with the literature are discussed, followed by more focused testing with cyclic loads and high displacement rates. Also, the mechanical characterization results are presented. 

### 3.1. Dynamic Mechanical Compression Test Results of Calcaneal Heel Pad Surrogates 

**Figure 4:**
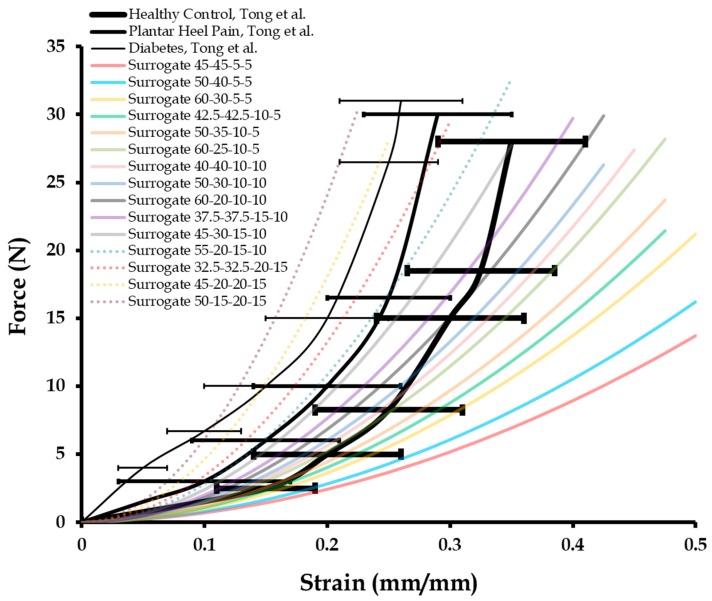
Force versus strain for the four-part fat pad surrogate compositions (in wt %) compared with that of literature results on subjects grouped as healthy control, plantar heel pain, and diabetes. Literature results were reproduced from Tong et al. [16], Copyright 2003, with permission from Elsevier.

The 15 surrogate compositions were tested under uniaxial compression at a test rate of 0.08 mm/s in order to compare with the literature results [16]. Specifically, the force versus strain plots corresponding to the biomechanical behavior of healthy calcaneal heel pads tested in vivo were reproduced (Figure 4). Also, similar plots for calcaneal heel pads tested in vivo for patients with plantar pain and diabetes were reproduced (Figure 4). The average force versus strain response of 15 surrogate compositions, each tested three times with high repeatability (*R*^2^ > 0.95), are presented in Figure 4 along with the literature plots [16]. To identify surrogate compositions simulating the average mechanical behavior of the calcaneal heel pads of healthy subjects, ones with plantar pain, and diabetes, an *R*^2^ correlation analysis was performed between the three average literature plots, and each of the 15 surrogate plots in Figure 4. In each of these three analyses, 21 data points were used to estimate the *R*^2^ values. Table 2 presents the correlation quantities calculated, out of which, the 60-20-10-10, 55-20-15-10, and 45-20-20-15 surrogate compositions are found to exhibit the highest correlations with the healthy control (*R*^2^ = 0.92), plantar heel pain group (*R*^2^ = 0.94), and diabetes group (*R*^2^ = 0.86), respectively. As the error bars (standard deviations) in the literature data were not considered while conducting these correlations, it was important to identify surrogate ranges to represent calcaneal heel properties across subjects. Therefore, a correlation value of ≥0.8 was set as a threshold for deciding whether surrogate compositions were accurate enough to simulate calcaneal fat pad mechanical properties. For healthy fat pad surrogates, a four-part material composition with 50–60 wt % of part A (Shore 00-10), 20–30 wt % of part B (Shore 00-10), 10–15 wt % of part A (Shore 30A), and 10 wt % of part B (Shore 30A) was selected. Similarly, for calcaneal fat pad surrogates for plantar pain patients, the four-part composition identified was with 32.5–55 wt % of part A (Shore 00-10), 20–32.5 wt % of part B (Shore 00-10), 15–20 wt % of part A (Shore 30A), and 10–15 wt % of part B (Shore 30A). For calcaneal fat pad material properties across diabetes patients, the four-part surrogate composition selected was with 45–50 wt % of part A (Shore 00-10), 15–20 wt % of part B (Shore 00-10), 20 wt % of part A (Shore 30A), and 15 wt % of part B (Shore 30A). For further testing, the 60-20-10-10 composition was employed as the healthy control model. 

Some limitations of the dynamic test framework include the nonconsideration of shear, bending, and torsional loading conditions experienced in different gait activities. Also, the role of fatigue on the calcaneal heel pad surrogates needs to be investigated. With respect to the load application, for in vivo studies, the calcaneal fat pat interacts with other tissues and joint components (such as the calcaneus bone) within the entire limb. This was not considered in the current framework, and the surrogates were assumed to simulate the overall effect of such interactions. 

### 3.2. Cyclic Mechanical Compression Tests and Repeatibility of Calcaneal Heel Pad Surrogates

The control surrogate composition identified for the calcaneal heel pad of healthy subjects (60-20-10-10), was recreated to produce ten specimens with compositions of precisely measured 60 wt % of part A (Shore 00-10), 20 wt % of part B (Shore 00-10), 10 wt % of part A (Shore 30A), and 10 wt % of part B (Shore 30A). These ten test specimens were loaded and unloaded at a displacement rate of 1.96 mm/s for five cycles to understand their similarity with the literature-based in vivo tests by Fontanella et al. [10]. 

**Figure 5:**
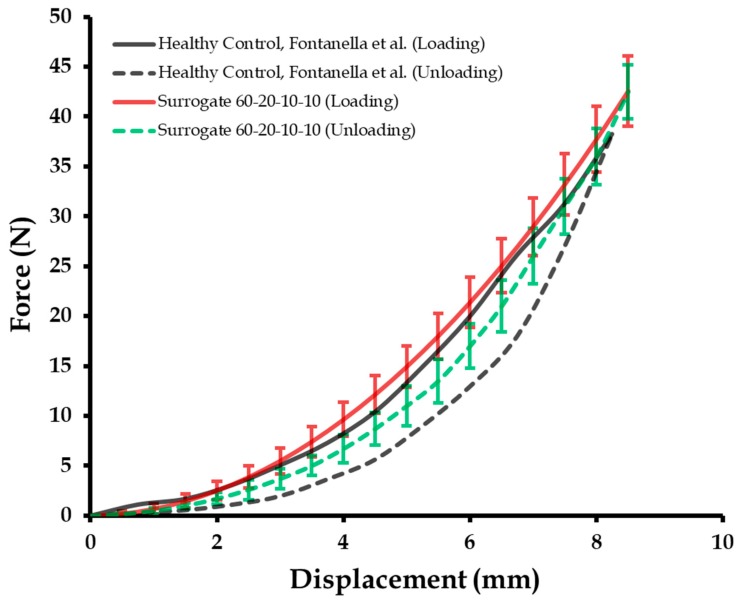
Cyclic force versus displacement of the control surrogate compared with the literature results (reproduced from Fontanella et al.[10], Copyright 2012, with permission from Elsevier).

Figure 5 captures the results (force versus displacement), where the surrogate response compared well with that of the literature-based loading curve [10]. An *R*^2^ value of 0.978 was estimated for the correlation index. Also, the loading tests were considered repeatable as the maximum variation was within ±10% of the maximum load [63]. For the unloading curve, the surrogate response is closer to the surrogate loading curve when compared to the analogous literature-based unloading tests (*R*^2^ = 0.693) [10]. This observation can be attributed with the higher viscoelasticity of elastomers than tissues, which allows for recovery with minimal permanent deformation. Also, the residual stresses observed in the calcaneal heel pad were absent in the elastomeric surrogates. With respect to repeatability of the unloading cycle, the maximum variation was also within ±10% of the maximum load [63]. The EDR, computed as the ratio between the area of the hysteretic cycle and the area under the loading curve (Figure 5), is 0.26 ± 0.03 for the literature plot [10]. The mean EDR estimated for the surrogate is approximately 0.22. It should be mentioned here that the calcaneal heel pad mechanical properties may change significantly with an increase in the number of loading cycles. However, the mechanical properties of the elastomeric surrogates may change minimally with time, as has been observed in several studies [30,32]. 

### 3.3. Compression Tests of Calcaneal Heel Pad Surrogates at High Displacement Rates

**Figure 6:**
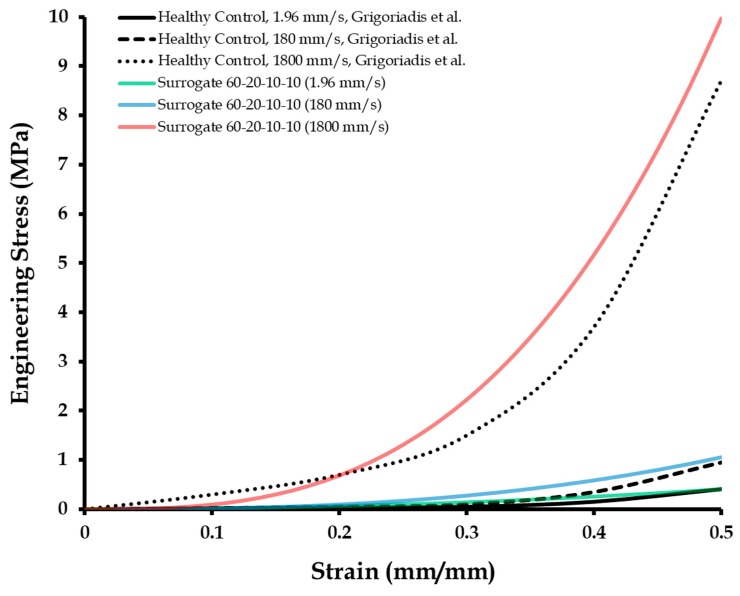
Engineering stress versus strain of the control surrogate at different displacement rates, compared with the literature [11], published under the Creative Commons Attribution (CC BY) license (http://creativecommons.org/licenses/by/4.0/).

High displacement rate testing was conducted on the healthy control surrogate composition (60-20-10-10), for comparison with the literature-based in vitro (cadaveric) tests [11]. The test rates employed were 1.96 mm/s, 180 mm/s (i.e., strain rate of 10/s), and 1800 mm/s (i.e., strain rate of 100/s). Figure 6 presents the results. The surrogate behaved very similar (*R*^2^ = 0.97) to the calcaneal heel pad tested at the low displacement rate of 1.96 mm/s. However, at the higher displacement rate of 180 mm/s, often experienced in walking or running [11], the mechanical response of the surrogate was higher (*R*^2^ = 0.86) than that of the in vitro literature results. For very high test rates of 1800 mm/s, the mechanical behavior of the surrogate was more stiff than that of the calcaneal heel pad tested in vitro in the literature [11]. The cause of this observation is unknown and could be a possible effect of material anisotropy (due to the honeycomb structure of the macrochambers) of the calcaneal pad, which will be investigated in future studies. The degree of correlation between the surrogate and literature results at the 1800 mm/s test rate was 0.82. As the correlation between the surrogates and literature data were all found to be above the value of 0.8, the surrogates were considered to be accurate for simulating high displacement or strain rate effects. 

Hyperelastic material characterization using Yeoh’s model was conducted on the stress versus strain responses of the control surrogate tested at the three different displacement rates (1.96, 180, and 1800 mm/s). Table 3 lists the Yeoh’s material parameters ($c_{1}$, $c_{2}$, and $c_{3}$) corresponding to each test rate. It should be mentioned here that as the Yeoh’s hyperelastic model does not consider displacement or strain rate effects, a different set of hyperelastic parameters were estimated for the same control surrogate tested at each different displacement rate. Apart from the control surrogate characterization, hyperelastic coefficients were also quantified for the 55-20-15-10 and 45-20-20-15 surrogates (tested at 1.96 mm/s), corresponding to the calcaneal heel pad properties for subjects with plantar pain and diabetes, respectively. These material parameters can be used for applications in computational modeling of the foot. Also, these numerical results can help in testing and validation of the developed calcaneal heel pad surrogates in future studies. A limitation of the material characterization framework which should be acknowledged here is that it did not take into account displacement rate effects, and thus different characterization parameters were estimated for the same surrogate tested at different displacement rates.

## 4. Conclusions

In this work, biofidelic surrogates were developed to simulate the in vivo and in vitro compressive mechanical properties of the calcaneal heel pad in healthy and diseased conditions (i.e., plantar fasciitis and diabetes). A four-part elastomeric material system was used to fabricate the surrogates, and their mechanical properties were compared with the literature-based in vivo dynamic and cyclic load testing, and in vitro (cadaveric) high strain rate tests. The surrogates were comparable with the calcaneal fat pad with a degree of correlation greater than 0.8 in all tests. Integration of these biofidelic surrogates with a prosthetic foot model will allow for the investigation of the effect of fat pad material property on plantar shock absorption and foot pain pathologies, for different simulated gait activities. Additionally, such a foot surrogate model will be useful for the experimental testing of shock absorption characteristics of preventive orthoses.

## Figures and Tables

**Table 1:** Four-part elastomer compositions (in wt %) tested for fabricating the calcaneal fat pad surrogates.

Test Specimen No.	Shore 00-10		Shore 30A	
	Part A	Part B	Part A	Part B
1	45	45	5	5
2	50	40	5	5
3	60	30	5	5
4	42.5	42.5	10	5
5	50	35	10	5
6	60	25	10	5
7	40	40	10	10
8	50	30	10	10
9	60	20	10	10
10	37.5	37.5	15	10
11	45	30	15	10
12	55	20	15	10
13	32.5	32.5	20	15
14	45	20	20	15
15	50	15	20	15

**Table 2:** *R*-squared correlations of mechanical properties of surrogates compared with average calcaneal heel pad properties recorded in vivo in the literature (reproduced from Tong et al. [16], Copyright 2003, with permission from Elsevier).

Surrogate Composition	Healthy Control, Tong et al.	Plantar Heel Pain, Tong et al.	Diabetes, Tong et al.
45-45-5-5	0.32	0.17	0.02
50-40-5-5	0.36	0.20	0.05
60-30-5-5	0.39	0.24	0.10
42.5-42.5-10-5	0.53	0.28	0.13
50-35-10-5	0.64	0.31	0.16
60-25-10-5	0.66	0.36	0.19
40-40-10-10	0.71	0.42	0.24
50-30-10-10	0.85	0.47	0.42
60-20-10-10 ^a^	0.92 ^a^	0.53	0.49
37.5-37.5-15-10	0.87	0.68	0.63
45-30-15-10	0.73	0.87	0.66
55-20-15-10 ^a^	0.69	0.94 ^a^	0.71
32.5-32.5-20-15	0.55	0.88	0.78
45-20-20-15 ^a^	0.38	0.73	0.86 ^a^
50-15-20-15	0.22	0.66	0.80

^a^ Highest correlation in each group.

**Table 3:** Yeoh’s material parameters for calcaneal heel pad surrogates tested at different displacement rates, and also for surrogates simulating plantar pain and diabetes.

Surrogate	$c_{1}(\mathrm{MPa})$	$c_{2}(\mathrm{MPa})$	$c_{3}(\mathrm{MPa})$
60-20-10-10 (1.96 mm/s)	0.0002	0.0090	0.0080
60-20-10-10 (180 mm/s)	0.0050	0.0100	0.0120
60-20-10-10 (1800 mm/s)	0.7200	0.3500	0.8800
Plantar pain (1.96 mm/s)	0.0004	0.0090	0.0100
Diabetes (1.96 mm/min)	0.0006	0.0150	0.0100
